# Palatal development of preterm and low birthweight infants compared to term infants – What do we know? Part 1: The palate of the term newborn

**DOI:** 10.1186/1746-160X-1-8

**Published:** 2005-10-28

**Authors:** Ariane Hohoff, Heike Rabe, Ulrike Ehmer, Erik Harms

**Affiliations:** 1 Poliklinik für Kieferorthopädie, Universitätsklinikum, Westfälische Wilhelms-Universität, Münster, Germany; 2 Department of Neonatology, Brighton & Sussex University Hospitals, UK; 3 Klinik für Kinderheilkunde, Division of Neonatology, Universitätsklinikum, Westfälische Wilhelms-Universität, Münster, Germany

## Abstract

**Background:**

The evidence on prematurity as 'a priori' a risk for palatal disturbances that increase the need for orthodontic or orthognathic treatment is still weak. Further well-designed clinical studies are needed. The objective of this review is to provide a fundamental analysis of methodologies, confounding factors, and outcomes of studies on palatal development. One focus of this review is the analysis of studies on the palate of the term newborn, since knowing what is 'normal' is a precondition of being able to assess abnormalities.

**Methods:**

A search profile based on Cochrane search strategies applied to 10 medical databases was used to identify existing studies. Articles, mainly those published before 1960, were identified from hand searches in textbooks, encyclopedias, reference lists and bibliographies. Sources in English, German, and French of more than a century were included. Data for term infants were recalculated if particular information about weight, length, or maturity was given. The extracted values, especially those from non-English paper sources, were provided unfiltered for comparison.

**Results:**

The search strategy yielded 182 articles, of which 155 articles remained for final analysis. Morphology of the term newborn's palate was of great interest in the first half of the last century. Two general methodologies were used to assess palatal morphology: visual and metrical descriptions. Most of the studies on term infants suffer from lack of reliability tests. The groove system was recognized as the distinctive feature of the infant palate. The shape of the palate of the term infant may vary considerably, both visually and metrically. Gender, race, mode of delivery, and nasal deformities were identified as causes contributing to altered palatal morphology. Until today, anatomical features of the newborn's palate are subject to a non-uniform nomenclature.

**Conclusion:**

Today's knowledge of a newborn's 'normal' palatal morphology is based on non-standardized and limited methodologies for measuring a three-dimensional shape. This shortcoming increases bias and is the reason for contradictory research results, especially if pathologic conditions like syndromes or prematurity are involved. Adequate measurement techniques are needed and the 'normal palatal morphology' should be defined prior to new clinical studies on palatal development.

## Background

Preterm infants, i.e. those born before completion of 37 gestational weeks, account for 6–10% of births in Western society [1-3]. Preterm infants form the majority of low birthweight infants [3]. By definition, neonates weighing less than 2500 g are described as low birthweight infants [4]. The proportion of neonates weighing less than 1500 g (very low birthweight) is approximally 1–1.5% of all newborns [3]. As preterm infants <1500 – 1800 g and <32*nd *– 34*th *gestational week have an insufficiently developed sucking response, they normally have to be fed through an orogastric tube.

The factors discussed as potential triggers of a premature birth include: high or low age of the mother, low socio-economic status, inadequate antenatal care, drug, alcohol and nicotine abuse, diabetes, multiple pregnancies [5], anemia, previous miscarriages or abortions, deformity of the uterus, abnormal presentation of the fetus, endocrine disorders, excessive mental or physical strain on the pregnant woman [6], stress [7], hypertension [8], and infections [9]. The potential influence of periodontal infections in the mother on the risk of a premature birth is a matter on which there is no general agreement: Many studies report an increased risk [10-14] whereas others found no evidence of such an association [15].

Preterm infants suffer not only from the effects of a shorter antenatal development period but from the immaturity of their organs [4], involving the risk of neonatal complications such as immaturity of the liver and kidney vitamin d metabolism [16,17], pulmonary diseases, hyperbilirubinemia and hypocalcemia [18] as well as respiratory distress, apnea, hypoglycemia, cardiac defects, infections, metabolic bone diseases and intracranial hemorrhages [19]. The latter have a significant impact on the function of rooting, non-nutritive sucking and suck-swallow responses [20].

Longitudinal studies confirm that the physical and cognitive development of most VLBW infants is delayed [21-24]. The median incidence of cerebral palsy is 7.7%, and that of disability 25% [23]. Real chances of survival without a substantially impaired state of health are not to be expected before completion of the 25*th *– 26*th *gestational week. Although substantial health impairment is to be expected in 2/3 to 3/4 of infants born in the 24*th *gestational week, the survival rate is meanwhile 50 – 60%, and in those born in the 25*th *GW as high as 70 – 80% [3].

With the survival prospects of preterm infants having undergone such a dramatic improvement [2,25-29], research into the development of these small patients can and must now be extended beyond securing their mere survival to other areas such as their physical and cognitive development. The morbidity potential associated with the premature birth needs to be investigated [30-32]. Only if the problems resulting from premature birth are exactly known preventive measures can be taken.

**Figure 1:**
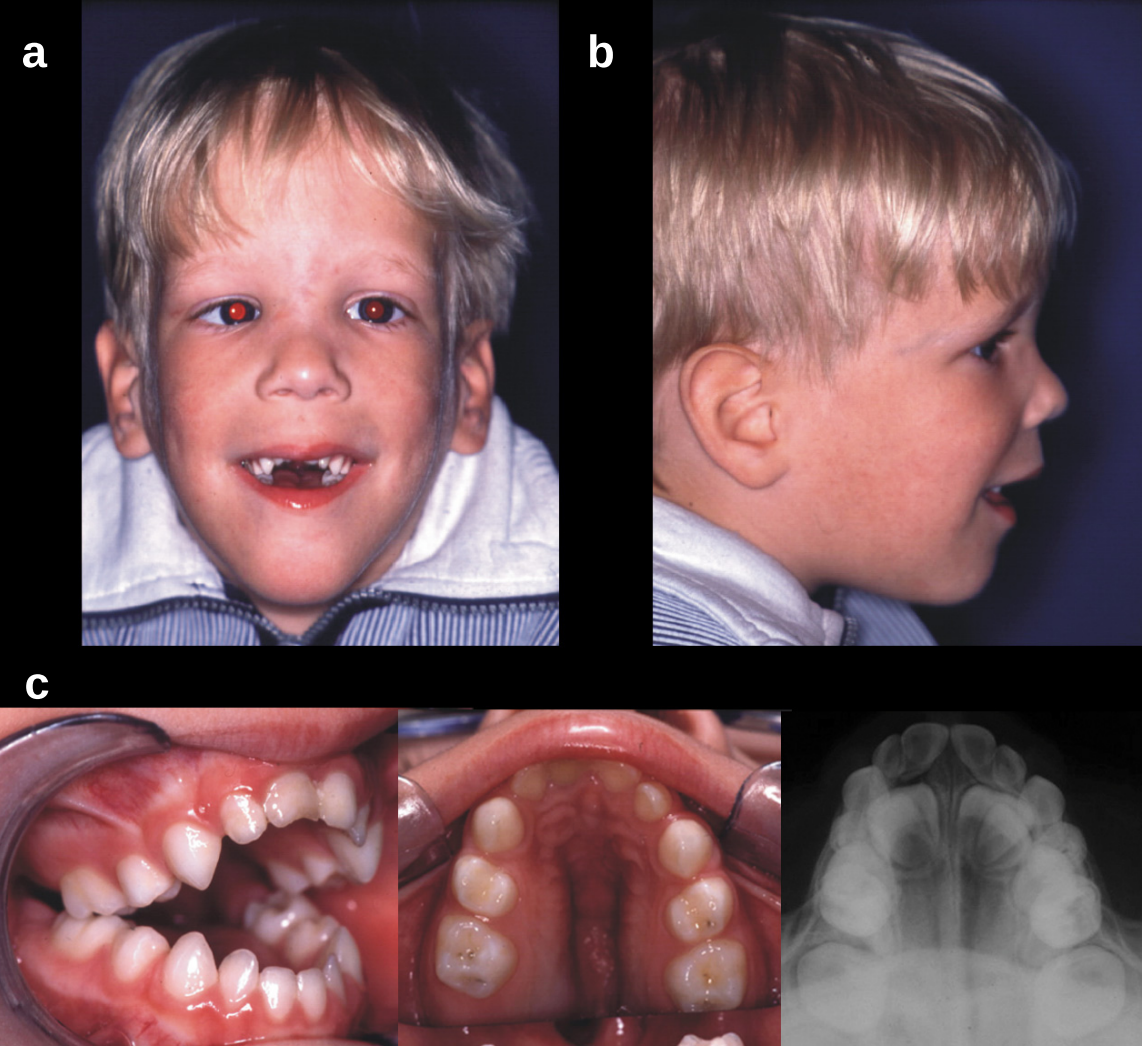
**a-e **Facial appearance (a, b), circular open bite (c) and palatal aspect (d) of a postnatally orotracheally intubated preterm infant in the initial phase of the dentition. Notice that the teeth of the child are '*in occlusion*' on Figures a-c. The food intake – limited to soft or mashed foods due to the extreme dysgnathia – leads to marked frustration. The infant is teased because of its eating problems and the shape of its jaw and head. Radiography revealed premature ossification of the median suture (e).

The orofacial region plays an important role in the infant's development in general: the mouth has been described as the '*cockpit of the awareness of the term infant and of its most discriminate responses*' [33]. However, in the early stages of the development of the oral cavity, the soft bones of the palate are malleable and pressure from any object can easily mould the shape of the palate [34]. Thus, at an early stage the palate in particular may be subject to influences such as mode of delivery [35], positioning and gravitational forces [2], oral intubation, sucking respectively inadequate sucking response, delayed primary tooth eruption or general hypotonia and its development may in turn affect the infant's food intake, breathing, phonation, dental development, facial appearance [2], esthetics and psychosocial development (Figure 1a–e).

The evidence of these consequences is still weak. A recent published systematic review [36] could not answer the questions on whether premature birth causes permanent alteration of palatal morphology, alteration of dental occlusion, and altered tooth-crown dimensions. The scientific evidence was too weak because of the contradictory results and lack of longitudinal studies.

Systematic reviews are not subject to the weakness of conventional narrative literature reviews because of the defined methods used to identify and reject studies; therefore, the conclusions are more reliable. However, systematic reviews are also open to questioning. The main issue to which criticism is addressed is the oversimplification of results by focusing on overall effects and downplaying mediating effects [37]. Contradictory results and methodological heterogeneity are common problems in the constitution of a systematic review. Six out of seven recently published systematic reviews (PubMed search: 'systematic review' AND orthod*) [36,38-43] concluded – irrespective of the research question – that further well-designed studies are needed.

It is therefore necessary to provide prospective investigators with methodological details of previous studies, especially in the field of morphometric assessment of palatal development, where new and more accurate methods have been established in the recent past. Moreover, information in different languages and without a restriction to particular databases and time periods must be included – a precondition which has not been considered yet.

The objective of this review is to provide a fundamental analysis of methodologies, confounding factors, and outcomes of studies on palatal development of preterm and low birthweight infants as compared to term infants. This review will be a major source of unfiltered data from more than a century, including also literature in German and French.

## Methods

The research was conducted according to the proposals of Greenhalgh [44,45] and the search strategies of the Cochrane Oral Health Group, the Cochrane Neonatal Group, the Cochrane Pregnancy Group and the Cochrane Childbirth Group were applied. As a recent paper pointed out the truncation of orthodontic* as suggested by the Cochrane Oral Health group to be entailing the implicit exclusion of relevant articles [46] other search strategies were used in addition to those of the Cochrane groups: (((child* OR infant* OR (low birthweight) OR neonate* OR premature* OR preterm*) AND (alveol* OR gum* OR palate* OR maxill* OR orthodon* OR groov*)) NOT (syndrom* OR cleft* OR cancer* OR carcino* OR fract* OR traum* OR surg* OR infect* OR occlusion* OR malocclusion* OR laser* OR (orthodontic treatment) OR caries OR lung OR cell OR cancer [sb] OR space [sb] OR cam [sb] OR tox [sb] OR history [sb] OR aids [sb] OR letter [pt])) Field: Title/Abstract, Limits: All Child: 0–18 years, Human.

Electronic literature research comprised the following medical databases: AMED, BIOSIS, CareLit, Cochrane Library, Current Contents, EMBASE, KindHeilk, Oldmedline, Pubmed, Web of Science. Additionally, 'hand search' was performed in text books and encyclopedias relevant to the subject, and in the following journals, including supplements and abstract bands: Clinics in Perinatology, vol. 21–30 (1994–2003); Der Kinderarzt vol. 16–21 (1985–1990) and vol. 25–31 (1994–200); Der Kinder- und Jugendarzt vol. 32–33 (2001–2002); Kinderärztliche Praxis vol. 53–61 (1985–1993) and 68–73 (1997–2002); Pediatric Clinics vol. 32–50 (1985–2003); Pediatric Research vol. 19–33:1 (1985–1993), 33:3–52 (1995–2002) and Pediatrics vol. 75–110 (1985–2002). Retrieved publications were checked for references and, where appropiate, these publications found in the bibliographies were considered in the review.

### Selection criteria

Sources in English, German and French were included from 1900 to 1/2004: non-metric visual findings, metric studies with intraoral measurements if no absolute numerical data were given but only visual findings were expressed in relative terms, and metric studies on plaster casts made from impressions of the palate. Only data provided in the reports were considered, and no attempt was made to contact the authors for missing or 'raw' data, because our research reached back to the beginning of the last century so that it would not have been possible to contact all authors.

As the authors of the review had been strongly involved in the subject matter, it was not possible to 'blind' them for the studies. Titles and/or abstracts of all citations were screened by one author (AH). In cases of doubt, the inclusion or exclusion of studies was discussed, and consensus was reached, among all authors. The full text of all relevant studies was evaluated. Exclusion criteria and affected studies are listed in Table 1 (see Additional file 1).

## Results

The electronic search strategy resulted in 141 articles, six abstracts, four conference papers, eight letters, six dissertations, and two masters theses. By hand search, eight abstracts, six bookchapters, and one encyclopedia were identified. Twenty-eight studies were excluded (Table 1, see Additional file 1) and one hundred fifty-five articles remained for final analysis. Among these, one hundred nineteen studies assessed morphometrically the development of the palate.

The first identified study was published in 1934 [47]. Looking at the different publication years and the different research questions, the articles can be divided into two parts: morphology of the preterm palate and morphology of the term newborn palate. The latter research topic was of great interest before 1960, whereas research on the preterm palate had its peak in 1985.

A further pattern to classify the research is the general methodology used for morphological assessments. Two different approaches could be identified for both groups, term and preterm infants: visual descriptions and metrical descriptions of the palatal configuration. Therefore, the review presented here follows the given patterns of past research and is divided into two parts.

Part 1 summarizes the applied descriptions of the palate in the term newborn. Without knowledge of the term infant's normal oral structures, it would be impossible to recognize abnormalities in the preterm infant's palate. The review of papers and bookchapters using visual descriptions of the term infant's palate – which are important for a general overview for the clinician – is followed by a presentation of metrical studies, which are necessary to validate clinical impressions which are the major source for measurements. The analysis of the studies is therefore ordered as follows.

• Visual description of the palatal configuration of the term newborn

- Palatal configuration with respect to gender and race

• Metric description of the palatal configuration of fullterm infants

- Palatal configuration with respect to gender and race

- Palatal configuration with respect to cranial index

- Palatal configuration with respect to mode of delivery

- Palatal configuration with respect to nasal deformities

### Visual description of the palatal configuration of the term newborn

In the newborn, the jaw already displays the palatine rugae present in the adult as well as the frenulum and the incisive papilla. In most cases the frenulum, which is located between the lip and the incisive papilla, recedes. If it fails to do so, it is known as a persisting tectolabial frenulum, which may later give rise to a midline diastema [48].

**Figure 2:**
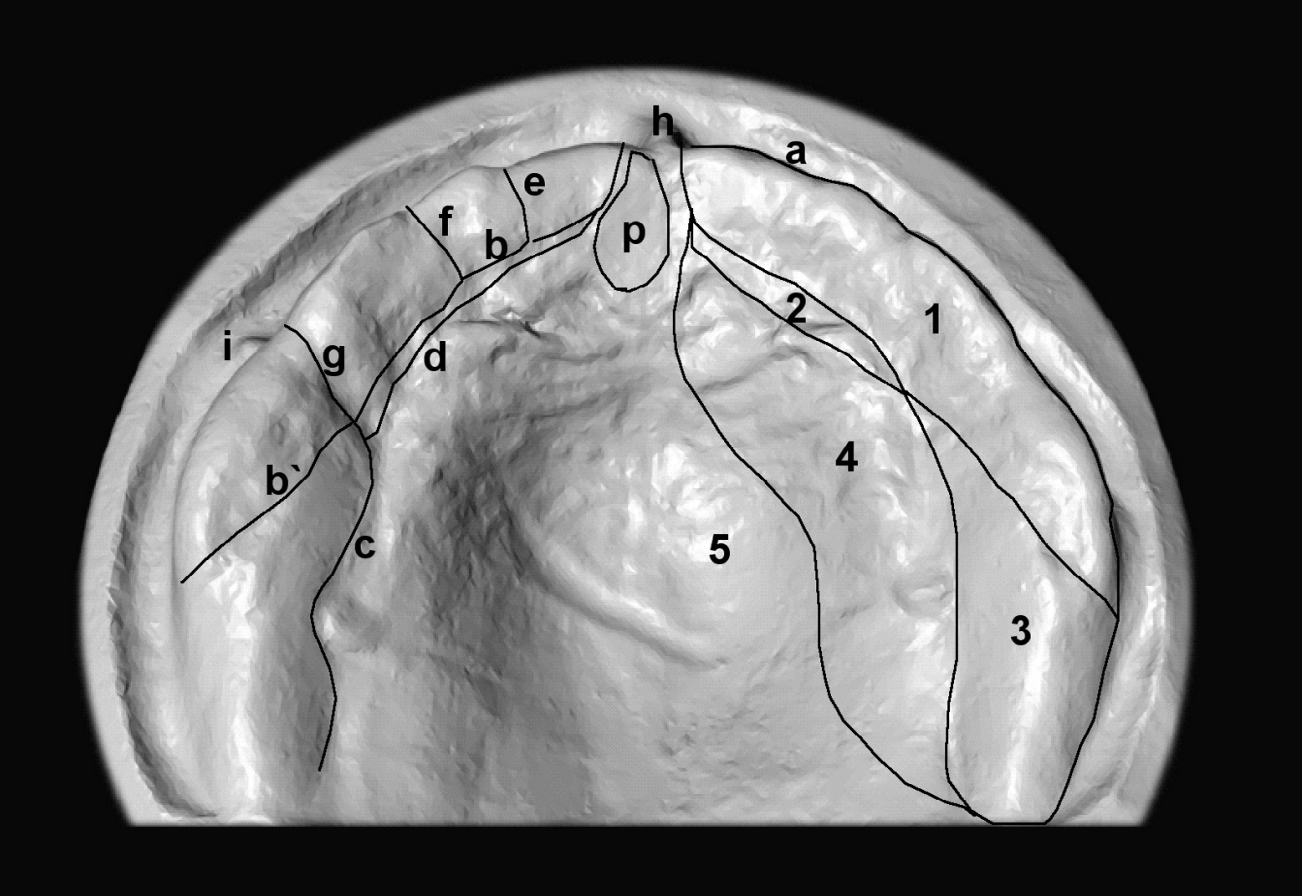
The alveolar portion of the upper gum pad is separated from the palate by a groove. The alveolar portion itself is again divided into buccal and lingual portions which are also separated by grooves. The former is the larger, participates in the formation of the sheath and socket of the teeth, and is further divided by transverse grooves or sulci into segments corresponding to the developing tooth germs. For nomenclature of palatal structures, see Table 2 (Additional file 2). Interestingly, the 'Terminologia Anatomica' contains for discription of palatal structures only the following terms: Frenulum labii superioris (Frenulum of upper lip); Palatum (Palate); Palatum durum (Hard palate); Palatum molle, Velum palatinum (Soft palate); Raphe palati (Palatine raphe); Plicae palatinae transversae, Rugae palatinae (Transverse palatine folds; Palatine rugae); Papilla incisiva (Incisive papilla).

In addition to the structures present in the adult, however, the maxilla of the newborn is characterized by a special feature: the groove system (Figure 2). This separates clearly visible maxillofacial regions and valla from one another and, like the frenula, is subject to a non-uniform nomenclature [49]. According to an investigation on palatal casts of 500 newborn fullterm children, the maxillary alveolar arch is marked along its whole length by the dental groove which divides it into two parts, a lateral labiobuccal and a medial lingual portion; it is through the former of these that the teeth eventually erupt [50] (Figure 2). The gum pad is divided into ten segments [51] which correspond to the developing tooth germs [50]. The central incisor and canine segments are approximately equal in size and are well marked; they are separated from the smaller lateral incisor segment, which is indistinct and sometimes lies lingual to them, by two shallow grooves. The lateral sulcus runs anteriorly from the lingual to the labial aspects and sometimes extends to a lateral frenum, this sulcus is the anterior margin of the first deciduous molar segments, which are the largest [51]. The second molar segment is more difficult to recognize. Merging with the dental groove, it can be made out lying somewhat lingual to the first molar segment. The gum is solid and firm throughout. In the distal part of the maxilla, the pseudoalveolar ridge can be recognized, a transient structure, which disappears in the first months of life (Figure 2).

What is of greatest importance within the framework of the present review is – as discussed in Part 3: 'consequences of intubation' – the most palatally located vallum (synonyms: tectal vallum, tectal ridge, lateral palatine ridge, lateral alveolar ridge, lateral palatine prominences or lateral palatine processes; see Figure 2, Table 2 (see Additional file 2)), which is a normal structure in the neonate and does not have an osseous but rather a connective-tissue base [52]. Hanson et al. reported after examining three deceased and 260 normal infants [52]: '*During early fetal life the lateral palatine ridges are composed of loose mesenchymal tissue with collagenous fibers embedded in lightly PAS-positive matrix. Alcian blue staining confirms the presence of acis mucopoloysaccharide. As development progresses the connective tissue become more dense, and the ridges appear less prominent in relation to the adjacent structures. As a result of the smoothing of the palatal vault and continued growth of the alveolar ridge, the lateral palatine ridges are less prominent in the normal full-term infant than in earlier stages.*'

Although there are obvious growth changes, the gum pad shows similar features at six months of age [25] (Table 3, see Additional file 3). Probably due to tongue thrust into the palatine vault [53], there is then a marked flattening of the lateral palatine ridges in the second year of life [52]. In the vast majority of the normal children (48 out of 56) the lateral palatine ridges are no longer apparent at the age of five years [52]. The configuration of the palate is then similar to that observed in adults.

Klemke [54] reported in his study on 200 newborns various kinds of upper jaws: a nearly semicircular form, a shape with a flattened anterior part and a nearly eleptic arch (percentages not given) (Table 3, see Additional file 3). In accordance, Neumann [55] described individual variations in palatal shape of 200 newborns, the majority of children, however, having horse-shoe or u-shaped palates (no percentages given) (Table 3, see Additional file 3). Approximately 1/3 of her probands presented a parable shape. Ott [56] diagnosed characteristic changes with respect to palatal shape in the course of time: up to the age of twelve months the majority of jaws had a semicircular anterior form with convergent sides, from 16 to 24 months parallel sides, and from 28 to 32 months divergent sides. She interpreted those changes in connection with the tooth eruption. The reliability of the method was not given in all three dissertations [54-56] (Table 3, see Additional file 3).

All elements of the bony palate are present in the fullterm neonate. The median palatine suture is a firm, fibrous articulation without fusion. The transverse suture between the palatine process of the maxilla and the intermaxillary bone is usually open and closes during the first year [57]. The palate during the first year of life is relatively broad and flat [58].

Epstein's Pearls, currently called palatal cysts [59] are remnants of epithelial tissue trapped during the palatal fusion. Their general incidence has been reported to be around 65% in full term newborns [60,61]. Bohn's nodules are remnants of mucuous gland tissue found on the buccal or lingual aspects of the dental ridges, dental lamina cysts (glands of Serres) are found along the crest of the alveolar ridges, both together are currently called alveolar cysts, and have also been referred to as '*gingival cysts*' or '*inclusion cysts*' [59]. An incidence of 36% of maxillary alveolar cysts in 1 – 5 days old full term newborns is reported [60]. The clinical description of palatal and alveolar cysts varies in color from white, to gray to yellow nodules, in size from a pinhead to 3 *mm*, and in numbers from 1 to 6 [59].

#### Palatal configuration with respect to gender and race

According to Dittrich [62] in newborn infants of both sexes the predominant form of the upper jaw is that with semicircular anterior parts and converging sides (male: 62%, female: 66%), followed by that with parallel sides (male: 18%, female: 2%). In contrast, Oelschlaegel [63] found a significant higher percentage of girls (62%) with a semicircular anterior part of the palate than boys (51%), and observed in boys among all possible forms of the side the parallel form to be the most frequent. In neither of the studies the reliability of the method was given, nor was mentioned explicitely that term infants had been examined. Leighton and Seshadri [64] found in a sample of 34 Caucasian full term infants at birth in only 14.7% midline notching of the upper gum pad compared to 34 matched Afro-Carribean infants, who had in 67.6% of the cases midline notching. The distribution of sexes in the sample was not given.

Huddart and Graf [65] also revealed differences between English, Italian and Swiss babies, affecting principally the anterior part of the upper gum pad and the contour of the palate. In a study with 500 normal full term newborns (82% blacks, 18% whites) from in the majority economically disadvantaged mothers aged less than 25 years in an urban setting (none of the babies had been admitted to the intensive care unit) a total of 21% alveolar notches was found. Those notches were significantly more common in blacks, with an odds ratio of 2.7 (a definition of the notches and their grades of severity was not given) [66]. There is speculation that notching is associated with a midline diastema in the primary and permanent dentition [61,67].

Friend et al. [66] found in 58% of the above mentioned sample cysts within the median raphe or the hard palate (Epstein pearls) or palatal cysts, which were defined by the authors as Bohn's nodules, i.e. whitish nodules at the junction of the hard and soft palate adjacent to the midpalate raphe (n.b. the different terminology in comparison to Donley et al. [59]). Most of the lesion in series were found at the hard-soft palate juncture. Midpalatal cysts were 2.5 times more likely to occur in white newborns (75%) than in blacks (55%). With respect to mentioned palatal structures, no gender difference was found.

Monteleone and McLellan [68] described results similar to those of Friend et al. [66]. The former found palatal cysts in 85% of the white children in contrast to 79% of the black children. With respect to palatal and alveolar cysts, Donley and Nelson [59] did not find significant differences between a cohort of caucasian children and a group of non-caucasian infants established from black, Latino and Indian children. Nor did they find gender differences.

Friend et al. [66] found no palatal cyst in the premaxillary region, all were posterior to the incisive foramen, which might be explained by the fact that the premaxilla is the first portion of the palate to fuse, if the pathogenesis of these lesions depends on entrapment of epithelium during fusion of the palatal shelves [69]. Alveolar cysts (grayish-white nodules along the crest of the alveolar mucosa or, less commonly on the lingual or facial borders) also were more likely in whites (26%) than in black children (11%, odds ratio 3.3, no distinction between upper and lower jaw was given).

Jorgenson et al. [61] found a higher total incidence in both races, but also diagnosed more alveolar cysts in whites (53%) than in blacks (40%). Although palatal and alveolar cysts are similar clinically and histologically, the former were more common, a discrepency which might be explained by the histopatholic presence of alveolar cysts in stillborns with lacking clinical manifestation [60].

Alveolar lymphangioma (blue, domed, fluid filled lesions on the alveolar ridges of either arcade, which occur typically bilaterally) was only found in black children, with teenage mothers at enhanced risk of having a child with this condition [66]. The latter authors found in none of the mentioned parameters a predeliction in gender, nor did Donley and Nelson [59].

### Metric description of the palatal configuration of fullterm infants

In order to validate clinical impressions, there is a need for measuring palates. Manufactureres would benefit from metric information to design save products [70].

Valid, plaster cast-based metric descriptions of the palatal configuration of healthy, term children around birth and in the first years of live are, however, rare (Table 3, see Additional file 3; Part 2: Table 4, see Additional file 4 of Part 2). Additionally, the following difficulties occur: in only four studies is named explicitely that term infants have been examined [25,50,67,71]; in two further studies, weight and/or maturity of the included children were given, enabling the authors of the review to recalculate the data for term infants; only eight studies [25,51,54,55,58,59,67,71] provided data on the weight and/or body length of the probands. Thus, the comparability of the results is limited.

The form of the upper jaw can considerably vary [54]. By recalculation of original data for term infants a correlation between maximum palatal width and weight, length of body, and biggest head circumference could be found (p < .05) [54].

In contrast, Leighton observed a low correlation (r = 0.4) between the dental arch width of neonates and their birthweight [67]. He moreover detected in a comparison of monozygotic twins, dizygotic same-sex twins and dizygotic different-sex twins that the differences in palatal width were twice as large in the last group as in the first. The author interpreted this as an indication that palatal width is genetically determined. Although genetic influence was clearly an important factor in determining gum pad morphology, there was only a weak correlation of size between the contained deciduous tooth crowns and the upper gum pad in the newborn [67], which may be due to the thick pad of fibrous tissue overlaying the developing tooth germ [25]. At the age of six months, however, the sum of the upper tooth diameters correlates significantly with maximum palatal width and postgingival width, as does weight. The size of the alveolar process is more linked to tooth size, whereas the total size of the gum pad, and the palate in particular, is more closely related to bodyweight. Sucking habits show a small but significant correlation with the stated palatal parameters, too, suggesting that a sucking habit is associated with narrowing of the palate, without its area or anterior length being altered. Age showed no significant correlation. In addition, the twin-based research revealed a significant hereditary influence on palatal width at birth [64]. A significant heriditary influence on the postgingival width was determined in a comparison of approximately 6-month-old identical twins versus fraternal, dissimilar-sex twins, but not versus fraternal, same-sex twins [25].

In six infants a pronounced transversal growth in the first six months of life was described, wheras a sagittal growth about zero was reported [72]. In contrast, in a study with 428 children, maximum palatal width and maximum palatal length grew at about the same rate in the first year, the width-length index remaining unchanged during the first year. Palatal height increased until 9 months, then remained quite constant up to the first year. The palatal dimensions varied widely during the first year, by about 7% for the width and length dimensions and by about 10 – 12% for maximum height (percentage variability means the coefficient of variation, i.e. the standard deviation divided by the means, times 100) [58]. A continuous increase in mean maximum palatal width of on average 7.45 *mm *from birth was measured (30.99 *mm*, value taken from [62]) to 32 months of age (38.44 *mm*), with the least changes occuring from 12 – 28 months [56]. The increase of mean length of the upper jaw was 9.18 *mm *(from 24.58 *mm *to 33.76 *mm*) (measurements from the beginning of the labial frenulum to a connecting line between the tubera).

#### Palatal configuration with respect to gender and race

The existence of ethnic differences could be important to those responsible for overseeing oral development of neonates in populations containing members of different ethnic groups.

Significant greater widths in the anterior part of the gum pad of 34 Afro-Carribean full term infants compared to 34 Caucasian full term subjects have been described [67]. No significant differences neither in the width of the gum pads distal to the lateral sulci nor in palatal height were detected. The differences in height only achieved statistical significance when expressed as the ratio of maximum width to palatal height. Unfortunately, the sex of the sample was not given so that the differences in size could have been wrongly attributed to races but could indeed have also occured due to gender differences: another study reveals the anterior parts of the upper arch of 7–12 years old girls to be smaller, but the posterior parts to be wider than those of the boys [73].

By recalculating the figures given by Neumann [55] no significant gender differences with respect to palatal width were found by the authors of the present review for spontaneously delivered term children aged 1–7 days, matched for birthweight and size (occipito-anterior vertex presentation exclusively). This is in contrast to the results given by the author herself, who found a significant sex difference for palatal width, but who did not distinguish between term and preterm children, included children up to three weeks of age, and did not consider the mode of presentation. Correlations between palatal width and size or birthweight could not be found, either, which is in accordance with the original results given by Neumann [55], and means that newborn children of the same size and birthweight can have differently dimensioned palates. The correlation coefficients for maximum palatal width and length with bodyweight and total body length in 100 male newborns were shown to be of low order (between 0.37 and 0.56), as the palatal dimensions are poorly correlated with each other and with other body dimensions. The palatal dimensions in the male are on average larger than in the female, corresponding to the larger mean size of male newborns [58].

Dittrich [62] and Oelschlägel [63] found significantly wider palates in boys compared to girls, the latter also significantly deeper palates in boys. Oelschlägel [63] did not find any gender differences for palatal length. In contrast, Dittrich measured significantly longer palates in boys [62]. This is in accordance with Hall et al. [48], who found the gender-related difference in palatal length to be accentuated with increasing age (reference values for palatal length, height and width are not given until the age of 5).

#### Palatal configuration with respect to cranial index

No correlation between cranial index (biparietal diameter in percentage of frontooccipital diameter) and palatal index (breadth in percentage of length) was found in 515 boys and 455 girls [63].

#### Palatal configuration with respect to mode of delivery

Every baby is subject to a certain amount of pressure during parturation, with head adaptations such as parietal bone and facial molding. Any molding of the face occurs across the maxilla, because the bimalar span is the widest part of the face. This molding compresses and deforms the soft maxilla, resulting in possible elevation of the arch of the palate [74].

No significant differences in palatal lengths, depths and widths dimensions between infants with spontaneous vertex presentation (n = 89, age 8 days, > 3.4 *kg*) and children with high or low forceps delivery (n = 10, age 8 days, > 3.4 *kg*) were described [51]. Klemke [54] and Hofbauer [75] did not detect an influence of mode of delivery on the jaws, either. Data for elective caesarian section and spontaneous face presentation was to small to draw any conclusions [51,55].

#### Palatal configuration with respect to nasal deformities

Kent et al. [71] tested the hypotheses made by Gray [76] that pressures in the maxilla during birth may cause elevation in one side of the palate and thus asymmetry of the hard palate which in turn could distort the vomer and septal cartilage. The former authors found no evidence of palatal asymmetry (Table 3, see Additional file 3) in 14 out of 500 children compared to 14 controls within three days of birth. The method of measurement was, however, quite coarse and the reliability of the method not given.

In contrast, at ages 3 – 6 years (n = 145) [74], 5 – 6 years (n = 145) and again in children '*aged about 8 years*' (n = 90) [77] statistically significant more often palatal asymmetries were found in children whose nasal septae were not in the midline at birth. In both studies, height of the palate was related neither to the evenness respectively unevenness of the palate nor to the type of septal deformity [74,77]. However, palatal asymmetry of width and height was present statistically significant most frequently in septae kinked to one side, less by septae deviated to both sides and least by straight septae. The method of measurement and the error of the method were not given in either of the two studies.

## List of abbreviations

[PT] preterm infant, [BW] birthweight, [LBW] low birthweight, [NBW] normal birthweight, [VLBW] very low birthweight, [NBW] normal birthweight, [GA] gestational age, [GW] gestational weeks, [NS] not significant

## Competing interests

The author(s) declare that they have no competing interests.

## Authors' contributions

AH designed the study, searched the databases, extracted the data, analyzed the results and wrote the manuscript. HR helped with study design, analysis and provided critical input in neonatal associated issues and revised the manuscript. UE and EH formulated the research question, helped with study design, analysis and in revising the manuscript. All authors read and approved the final manuscript.

## Supplementary Material

Additional File 1 Table 1. Excluded studies and reasons for exclusion. Click here for file

Additional File 3 Table 3. Studies on plaster casts. Click here for file

Additional File 2 Table 2. Nomenclature of palatal structures as presented in Figure 2. Click here for file
