# Supplementary material for: Palatal development of preterm and low birthweight infants compared to term infants – What do we know? Part 1: The palate of the term newborn
Source: Head Face Med. 2005 Oct 28;1:8. doi: 10.1186/1746-160X-1-8 (PMC1308841; doi:10.1186/1746-160X-1-8)
Supplement: Additional File 1 — Table 1. Excluded studies and reasons for exclusion. [file 1746-160X-1-8-S1.pdf]

Table 1. Excluded studies and reasons for exclusion.

Studies	Reasons for exclusion from review
[78]	Intraoral and therefore probably imprecise measurements, palatal depth calculated.
[79]	Measurements were taken directly in the mouth to the closest millimeter- too imprecise.
[80]	Reference points for measurements neither comparable to reference points of [92] nor to reference points of [97].
[81]	General remarks on growth.
[82]	Non- representative patients, undernourished, partially rachitic children.
[83]	Non- representative patients, undernourished, partially rachitic children.
[84]	No data given on number of children that have been examined.
[85]	Data based on dead body donations, though ‘no pathological material was used’ no information on reasons for death of the children were given.
[86]	Study on chimpanzee skulls.
[87]	Data given beginning at the third Y of live, not comparable to other studies.
[88]	No data given on reasons for deaths of body- donations, no data given on criteria for in- or exclusion.
[89]	Examination of 30 normal children at birth, but reference- points not comparable to other studies.
[90]	Examination of 30 normal children at birth, but reference- points not comparable to other studies.
[91]	No absolute values given (only information in indices without giving localisation of single measurements).
[93]	No data given on reasons for death of examined body- donations, no data given on inclusion and exclusion criteria or on differing numbers for each examination performed. No information concerning measuring method. Dental measurements were performed without giving information if and where edentulous jaws were measured.
[94]	Reference points for measurements neither comparable to reference points [92] nor to reference points of [97].
[95]	Data given beginning at the fifth Y of live, not comparable to other studies.
[96]	Data given beginning at the fifth Y of live, not comparable to other studies.
[98]	General remarks on growth.
[99]	General remarks on growth.
[100]	Eutrophic and dystrophic abortions and stillborns of various european countries without information on exclusion or inclusion of syndromes.
[49]	Eutrophic and dystrophic abortions and stillborns of various european countries without information on exclusion or inclusion of syndromes.
[101]	Data given on length values from spina nasalis anterior to spina nasalis posterior based on lateral cephs, no data given on palatal dimension.
	Subjects: 40 complete dry skulls of male infants died within 100 D of birth without any malformation, mean age 36 D.
[102]	Confounding of preterm and term infants.
[103]	Study on rats.
[104]	Intraoral and therefore probably imprecise measurements.
[105]	Data given without information if own measurements were performed or if data from the literature was included.
[106]	No figures given, only plotted values.

[D] = days; [Y] = year(s).
