# Supplementary material for: Palatal development of preterm and low birthweight infants compared to term infants – What do we know? Part 1: The palate of the term newborn
Source: Head Face Med. 2005 Oct 28;1:8. doi: 10.1186/1746-160X-1-8 (PMC1308841; doi:10.1186/1746-160X-1-8)
Supplement: Additional File 3 — Table 3. Studies on plaster casts. [file 1746-160X-1-8-S3.pdf]

Table 3. Studies on plaster casts. Due to missing data on childrens' degree of maturity at birth no valid assignment into a group of term infants could be performed with few exceptions [4, 80, 69*, 31, 87]. In two studies [4, 109] probably quite flexible impression trays and in one [109] wax was used as impression material, therefore inaccuracies of impressions, casts and measurements can not be excluded.

Studies	Patients/ inclusion / exclusion	Material / method + validity
[47]	– 90 newborn livingborn M of normal form and structure, born at Bellevue, offspring of the poorer classes with one exception (black) caucasians.	– Impressions (modelling compound), stone cast.<br>– Projections by dental pantograph.<br>– Plane of orientation determined by the incisive and postgingival points.<br>– Reliablity not given.
[58]	– Inclusion: 428 caucasian infants (305 m, 123 f) under one Y from the vicinity of Bellevue Hospital.<br>– Most infants healthy or suffering from minor upper respiratory infections, somewhat under weight and height as compared with infants from a better economic environment.<br>– Exclusion: rickets, syphilis or other disease, which might effect palatal shape.<br>– Cave: the authors stated: asymmetrical palates are not infrequently seen in infants, even during the newborn period, they are regularly associated with asymmetry of the head (Comment: thus, syndromes might not have been excluded.).	– Impressions (wax), casts.<br>– Height: measured with Stanton`s apparatus.<br>– Other dimensions: Calipers.<br>– Measuring method and reliablity not given.
[50]	– 500 newborn fullterm children < 8 D	– Impressions (Nadrag Impression compound).<br>– Measuring method and reliablity not given.
[72]	– 5 M, 1 F, within one week after birth and at 6 MO.	– Dental casts.<br>– Photographic analysis with a cartographic projection of 'high precision', measuration index which allows a three dimensional correction of growth and a simultaneous projection of a mm grid for reporting differences traced on the graphics.<br>– Reliability not given.
[62]	– 518 male 'newborns', 465 female 'newborns', 1- 4 D, from the region of Leipzig, regardless their way of delivery (vaginal, forceps or caesarian section).	– Trays made from Piacryl with stents- like impression material, which could not stay within the mouth until total cure.<br>– Subsequent cooling of the impression in cold water, pouring of plaster casts.<br>– Height measurement with palatometer (graduated wire with a perpendicular sliding rod) to drop a perpendicular on the connecting line between right and left alveolar.<br>– Width measurements on the widest extension of the middle of the alveolar crest in transversal direction.<br>– Visual description of palatal form.<br>– No reliability of either of the methods given.
[74]	– 145 individuals between 3 and 6 Y, (400 assessed and documented at birth, those studied = random selection of those available from that total list), 4 not included in dental assessment (no reason given why), 9 without adequate medical history.<br>– 33 severe deformities causing symptoms at birth (e.g. snuffling, poor feeding) were treated with corrective manipulation.	– Classification of three kinds of septae at birth as found by nasal inspection or passage of nasal testing struts:<br>– A: straight (42 % at birth).<br>– B: kinked septum deformed to one side only (31 % at birth).<br>– C: septum deviated to both sides (27 % at birth).<br>– At ages 3-.6 dental casts, photos (available on 114), medical examination (anterior rhinoscopy) and history (nine without adequate history) assessed separately and finally correlated with findings of septal configuration.<br>– Dental casts assessed independently by two people and final findings mutually agreed upon.<br>– Silicone based impression from dental casts, divided coronally in 3 places (along mesial margins

<table>
<tr><td></td><td></td><td>of second deciduous molars, first deciduous molars and cuspids), three segments were traced on graph paper and height and width from the midline were measured at the middle cut; one point was alloted initially for a normal cast, and points were added for a difference in height of the two sides and for a difference in width; points for each subject were totalled = total dental score.
– Statistics: χ^2 test.
– Reliability not given.</td></tr>
<tr><td>[77]</td><td>– 145 randomly selected babies from a pool of 400 who had been assessed and documented at birth were assessed when aged 5- 6 Y (primary dentition present).
– +
– 90 patients reassessed ‘when aged about 8 Y’</td><td>– Ear, nose, throat examination (assessment of internal alignment of nasal septum at investigation timepoints see Gray et al. (1982) [74], medical history.
– All had plaster casts; silicone-based impression from the dental cast was divided along the mesial margin of the first deciduous molars and the outline traced on graph paper for measurements and symmetry analysis.
– ‘Most’ had facial photographs.</td></tr>
<tr><td>[110]</td><td>– 121 normal australian children without orthodontic treatment (69 M, 52 F).</td><td>– Random selection.
– Impressions (hydrocolloide), stone casts.
– Subjective assessment of palatal height (‘shallow’, ‘normal’, ‘deep’) (2 examiners) +
– Measurement to the nearest 0.5 mm (palatal width with vernier calipers, palatal height with a profil gauge (one investigator).
– Reproducibilty of the method $p < 0.01$ (students t-test) (measurement of 10 randomly selected dental casts 3 D apart).</td></tr>
<tr><td>[71]</td><td>– ‘500 babies’, GA 40 W, mean BW 3.01 kg, ‘born consecutively’, examination within 3 D after birth, therefrom 14 with deviation of the septum (therefrom 11 delivered per vaginam, 2 per forceps, 1 per caesarean section)
+
– 14 control children, GA 40 W, 3,2 kg (‘matched closely for ethnic origin, sex, parity, pregnancy history, presentation, labour, delivery and BW’).</td><td>– 28 plaster casts, marked at the midline (from labial frenulum attachment to nasopalatine foramina (Remark of the authors: as the labial frenulum can be deviated during the impression procedure it is not a reliable point for determining the midline).
– Measurement: simple graticule.
– Validity: not given.
– Results: palates of control and test children symmetrical to within 1mm.
<table>
<tr><td>distance posterior to the crest of the alveolus in the midline (mm)</td><td>mean (SD) of children with deviated septum (n=14)</td><td>mean (SD) of children without deviated septum (n=14)</td></tr>
<tr><td>5</td><td>24.3 (0.9)</td><td>24.2 (1.2)</td></tr>
<tr><td>10</td><td>29.8 (1.5)</td><td>29.3 (1.1)</td></tr>
<tr><td>15</td><td>29.5 (1.6)</td><td>28.8 (1.5)</td></tr>
</table></td></tr>
<tr><td>[54]</td><td>– a) 200 newborns examined on the first D of life, further observed the following 8 D during their stay in hospital.
+
b) 60 casts from the collection of Korkhaus (see Tab. 7).</td><td>– Specially designed impression trays made from aluminium, plastic impression mass, bite registration.
– Written notices on age, mode of presentation, position of the mandible, type of bite, form of the jaw, horizontal and vertical distance between the frontal parts of the jaws, sucking, habits (dummy sucking, sleeping position).
– Casts a) for determination of bite and form of the jaws
– Casts b) for measurements.
– No information given neither on how measurements were performed nor on reliability of the method.</td></tr>
<tr><td>[67]</td><td>– Inclusion: 388 newborn children of the Y 1953, born in King’s College hospital.
– Exclusion: children born at home or children, or children of mothers whose condtition did not allow a transfer to another clinic within 7 D</td><td>– Dental casts.
– Measuring method and reliability not given.</td></tr>
</table>

[25]	– Series of cases studied longitudinally at King´s College Hospital Dental School, London (1952-1982)<br>– 109 normal, full term children (61 m, 48 f)	– Composite impressions, Casts (50:/ 50 mixture plaster of Paris and artificial stone).<br>– All linear measurements to the nearest 1/10 mm.<br>– Error of the measurement (Dahlbergs method): max. width: 0.20 mm; width at postgingivalia 0.79 mm.
[64]	– 34 full term Caucasian infants, 6.5 D (SD 3.5, range 1- 16), 5.754 lbs (SD 0.823)<br>– 34 full term Afro-Carribean infants, 4.9 D (SD 2.8, range 1- 10), 5.747 lbd (SD 0.773)	– Thermoplastic modelling compound (Velvex) impressions.<br>– Measurements: sliding dial calliper to the nearest 0.1 mm.<br>Error of linear measurements by double determination of 10 cases taken at random max 0.328 mm according to method of Dahlberg.
[55]	– 180 term and 20 PT newborns, aged 0- 21 D,<br>– Values presented in Tab. 6 refer to a recalculation of the authors for term, spontaneously delivered children (occipito- anterior vertex presentation) aged 1- 7 D exclusively	– Paladon trays, impression material 'Xantigen Bayer', formed with the finger on the tray and pulled through the ethyl alcohol flame in order to obtain a smooth surface, which was immediately cooled with water. This resulted in three layers and a more precise reproduction.<br>– As cooling with cold water could not be performed in the infants mouth, thus a distorsion of the impression material could not be avoided in some cases.<br>– Measuring method and reliablity not given.
[63]	– 526 M, 470 F newborns, 4- 48 H old, with „normal upper jaws', regardless their way of delivery (vaginal or caesarean section).<br>– Exclusion of clefts and children or children of mothers with syphilis	– Trays made from Piacryl, plastic impression material from Harvard and De Trey covered with a thin vaseline layer, cooled in water after impression taking<br>– Measurements with a measuring rod.<br>– Height measurement with a graduated wire with a perpendicular sliding rod to drop a perpendicular on the connecting line between right and left alveolar.<br>– Width measurements on the widest extension of the middle of the alveolar crest in transversal direction.<br>– Visual desription of palatal form<br>– No reliabilty of either of the methods given.
[56]	– Recall of the children examined by Dittrich (1959) [62] every four months.	– Plastic trays, thermoplastic stents- like impression material from Jacobson (Leipzig), which could not stay within the mouth until total cure because the children made a stand against the procedure; hereby the exactness of reproduction of all details was partly diminished. Subsequently the impression tray was cooled in cold water, and plaster casts were poured out.<br>– Width measurements on the widest extension of the middle of the alveolar crest in transversal direction.<br>– Visual desription of palatal form.<br>– High drop off rate.<br>– Width measurements on casts with a pair of compasses on the middle of the alveolar crest.<br>– Height measurements from the deepest vault of palate in the raphe median line with a palatometer according to [63] and [62].<br>– Reliability not given.
[111]	– 26 'healthy westfalian infants'.	– Alginate impressions.<br>– Plaster casts.<br>– Measurement with a 3 D laser digitizer (precision: 20 µm in a measuring volume of 50 mm^3)
[51]	– Out of 709 infants 118 (116 white, 2 black, all in good health), 1-11 D from Bellevue Hospital for indigents.	– Impressions with modeling compound on soft metal trays.<br>– Imprint of maxillary and mandibular gum pads with been wax.<br>– Measurements with a vernier caliper to the nearest 0.1 mm, depth measured with a depth gauge.<br>– Reliablity not given.

* Data recalculated by the authors of the present review for term infants. [BW] = birthweight, [D] = day(s), [F] = female, [GA] = gestational age, [GW] = gestational weeks, [H] = hour(s), [LBW] = low birthweight, [M] = male, [MO] = month(s), [NBW] = normal birthweight, [NS] = not significant, [PT] = preterm, [VLBW] = very low birthweight, [W] = weeks, [Y] = year(s)).
