# Supplementary material for: Palatal development of preterm and low birthweight infants compared to term infants – What do we know? Part 1: The palate of the term newborn
Source: Head Face Med. 2005 Oct 28;1:8. doi: 10.1186/1746-160X-1-8 (PMC1308841; doi:10.1186/1746-160X-1-8)
Supplement: Additional File 2 — Table 2. Nomenclature of palatal structures as presented in Figure 2. [file 1746-160X-1-8-S2.pdf]

Table 2. Nomenclature of palatal structures as presented in Figure 2.

structure	Term	Reference
**a**	Buccal vestibule	[49]
	Outer alveolar groove	Schwarz*
	Vestibular buccal vestibule	Schwarz*, [105]
	Lip groove, labial groove	[51]
	Labio- gingival groove	Norberg*
	Outer buccal margin	Freiband*
	Vestibular groove, bucco-marginal groove, labio-bucco-tectal groove, labio-tectal groove, labio-gingival groove	Bolk*
**b**	Alveo-palatinal sulcus	Ashley-Montagu*
	Dento-gingival groove	Bolk*, [105]
**b’**	Postero-lateral sulcus	Ashley-Montagu*
**b + b’**	Tooth germ groove	[108], [49]
	Inner alveolar groove, dental groove	[62], [54], [55], Schwarz*
	Dental groove; dental and gingival grooves are divided by the incisal papilla into lateral halves [18]	[50], [51]
**c**	Transitory palatal fold	[62], [55], Schwarz*
	Transitory palatal groove	[31], [48], Bolk*
**d**	Anterior palatal groove	[105]
	Gingival groove	[108]
**c + d**	Gingival groove (defines the inner alveolar margin; gingival and dental grooves are divided by the incisal papilla into lateral halves [18])	[50], [51]
	Lingual groove	Freiband *
**e**	Anterior sulcus	Ashley-Montagu*
	Anterior groove	[108]
**f**	Antero-lateral sulcus	Ashley-Montagu*
	Antero-lateral groove	[108]
**g**	Lateral sulcus	Ashley-Montagu*<br>[25]
	Bucco-tectal groove	Bolk*<br>[108]
**h**	Frenulum labii superioris	[49]
	Frenulum tecto-labiale	Bolk*
	Frenulum labio-tectale	Norberg*
	Frenulum labiale	[105]
	Frenum labii	[51]
**i**	Frenulum of cheek	[31]
	Frenulum laterale	[49], [105]
**p**	Papilla incisiva	[76], [31]
**1**	Alveolar wall/ -bulge	Schwarz*, [62], [108], [54], [105],
	Dental wall	Bolk*
	Palatal wall	Gegenbaur*
**2**	Tektal wall	Bolk*, [105]
**p + 2 + 4**	Tektal wall	[55]
**3**	Pseudo alveolar wall	Schwarz*, [62], [108], [54], [55],
	Dental molar wall	[105]
	Alveolar wall	Retzius*<br>Gegenbaur*
**4**	Tektal bulge	Schwarz*, [54], [105]
	Lateral palatine ridge	[52]
	Lateral palatine shelve	[107]
	Lateral alveolar ridge	[102]
	Lateral palatine prominences, Lateral palatine processes	[107]
**2 + 4**	Tektal wall	[108]
**5**	Palatal vault	[105]
**vestibular part of 1**	Vestibular germ bulge	Schwarz*
**1 + 3**	Border bulge	Schwarz*
	Border area, ona marginalis	Bolk*
	Gingival ridge	[85]
**4 + 5**	Tegmen pris	Bolk*

*Taken from [49]; no source given in the bibliography [49].
